# Potential effects of heavy metal pollution from a cement factory near Saudi Arabia’s largest green turtle rookery

**DOI:** 10.1007/s10661-022-10063-2

**Published:** 2022-05-24

**Authors:** Lyndsey K. Tanabe, Susana Carvalho, Vijayalaxmi Dasari, Areen Nasif, Kaitlyn A. O’Toole, Michael L. Berumen

**Affiliations:** Division of Biological and Environmental Science and Engineering, Red Sea Research Center, King Abdullah University of Science and Technology, Thuwal, 23955 Saudi Arabia

**Keywords:** Red Sea, Cement pollution, Endangered species, Environmental assessment, Sand contamination, Turtle nesting threats

## Abstract

Sand samples were collected from four beaches near a cement factory in Ras Baridi, north of Yanbu, which hosts the largest green turtle rookery in Saudi Arabia. Heavy metal concentrations (Cd, Pb, Fe, Cr, Ni, Se, Sb, As, and Cu) were measured at three different depths. For most elements, there were no significant differences in concentrations among depths; however, significant differences were found among the nesting beaches in Ras Baridi, which were likely influenced by the wind direction from the factory. Fe, Cr, Cu, and Ni had elevated contamination factor values, suggesting that the nesting beaches downwind and adjacent to the cement factory contained moderately contaminated sand. Given the possibility of heavy metals being absorbed through eggshells, there is a potential risk of heavy metal contamination in clutches laid in Ras Baridi. The rising threat to the local ecology in Saudi Arabia due to recent coastal developments for tourism projects highlights the importance of monitoring heavy metal concentrations over time.

**Supplementary Information:**

The online version contains supplementary material available at 10.1007/s10661-022-10063-2.

## Introduction

Cement production is a major source of environmental pollution, with the industry accounting for 5% of the global CO_2_ production (Andrew, 2018). The excavation and grinding of rocks during cement production yields particulate matter, which can be blown away by wind and released into the surrounding environment (Arimoro et al., 2021; Bluvshtein et al., 2011). Furthermore, cement factories produce several heavy metals including Zn, Cd, Mn, Cu, Cr, Pb, and As (Adejumo et al., 1994). Heavy metals are naturally occurring elements; however, some are known to cause harm to the environment above certain thresholds (Tchounwou et al., 2012). Heavy metals have been reported to affect cellular organelles and enzymes involved in metabolism, detoxification, and damage repair in biological systems (Bánfalvi, 2011; Wang & Shi, 2001). Many heavy metals produced by cement factories are known to be toxic to living organisms, even at low concentrations (Kabata-Pendias & Mukherjee, 2007).

Pollution poses a major threat to the survival of marine turtles (Lutcavage et al., 1997), which can accumulate heavy metals through food, water, and sediments (Martínez-López et al., 2021; Zaib-Un-Nisa et al., 2021). Therefore, turtles are potential bioindicators of heavy metal contamination because they are long-living vertebrates with large home ranges (Bruno et al., 2021; Zaib-Un-Nisa et al., 2021). Several studies have assessed heavy metal concentrations within stranded sea turtle tissues (Kaska et al., 2004; Sakai et al., 1995; 2000; Storelli & Marcotrigiano, 2000), yet fewer studies analyzed trace metal concentrations in the nesting beaches (e.g., Çelik et al., 2006). A study on olive ridley turtles (*Lepidochelys olivacea*) in India found that the concentrations of nine different heavy metals measured from hatchlings exceeded those measured from freshly laid eggs, suggesting that turtle embryos accumulated metals from the nesting beach sand during incubation (Sahoo et al., 1996). Similarly, green turtle hatchlings in Oman had higher metal concentrations than those in freshly laid egg yolk, suggesting that excess metal contamination was likely dependent on the concentration of the contaminated sand during incubation (Al-Rawahy et al., 2007). Lastly, other studies have concluded that among the metals found in high concentrations in critical reptile tissues, there are several metals of priority concern (Al, As, Cd, Cr(VI), Cu, Hg, Mn, Ni, and Pb) (Grillitsch & Schiesari, 2010). These elements are known or suspected to cause various serious health effects (e.g., cancer, reproductive and developmental disorders, immune function, and endocrine disruption) (Grillitsch & Schiesari, 2010; Nordberg et al., 2007).

Five of the world’s seven sea turtle species, all considered vulnerable, endangered, or critically endangered by the International Union of the Conservation of Nature Red List (IUCN, 2021), are found in the Red Sea. Of these five species, only hawksbill (*Eretmochelys imbricata*) and green turtles (*Chelonia mydas*) nest regularly throughout Saudi Arabia’s 1760 km coastline (Mancini et al., 2015; Shimada et al., 2021). The country’s largest rookeries for hawksbill and green turtles are Waqqadi Island and Ras Baridi, respectively (Pilcher & Al-Merghani, 2000; Shimada et al., 2021). Ras Baridi is a coastal stretch of turtle nesting beaches located near the Yanbu Cement Factory, which in the 1980s, emitted an estimated 120 tons of partially processed cement dust per day (MEPA, 1983). This dust was then carried by wind, accumulated on turtle nesting beaches in the area, and caused hatchling mortality, likely by preventing the diffusion of O_2_ and CO_2_ from the nest chamber (Pilcher, 1999).

Several studies in Saudi Arabia have assessed the impacts of cement factories on the environment (e.g., Al-Omran et al., 2011; Al-Oud et al., 2011; El-Sherbiny et al., 2019); however, none evaluated the possible impact of heavy metals from cement factories on a turtle nesting beach. The objectives of this study were threefold. The first objective was to assess how wind affects the dispersion of heavy metals at four nesting beaches in Ras Baridi, which varied in distance from the Yanbu Cement Factory. The spatial distribution of metals around cement factories depends on dust particle size, soil pH, soil type, and prevailing wind direction (Al-Khashman & Shawabkeh, 2006; El-Sherbiny et al., 2019; Gupta & Sharma, 2013). Owing to the influence of wind in carrying the dust and associated pollutants, it was hypothesized that concentrations of heavy metals would be higher downwind of the Yanbu Cement Factory compared to upwind. The second objective was to assess any differences in heavy metal concentrations at the sand surface, compared to 30 and 50 cm depths, corresponding to the average depth of hawksbill and green turtle nests, respectively. Studies have shown that surface sediments have higher heavy metal concentrations than sub-surfaces (Al-Mur et al., 2017; Bahram et al., 2021); therefore, it was hypothesized that heavy metal concentrations would be higher in surface samples than in those collected at 30 and 50 cm depths. The third objective was to evaluate the heavy metal concentrations and the resulting environmental contamination levels (based on commonly used pollution indices, including the contamination factor and geo-accumulation index (Hakanson, 1980; Muller, 1969)) between the nesting beach adjacent to the Yanbu Cement Factory in Ras Baridi and Rabigh Beach, the uncontaminated reference nesting site located 200 km south. A study conducted in Saudi Arabia found that the distribution of heavy metals in the surface soils around a cement factory was affected by both the cement industry and traffic emissions (El-Sherbiny et al., 2019); thus, it was hypothesized that the concentrations of heavy metals would be higher at the nesting site near the cement factory than the reference site farther from human impact.

## Methods

**Fig. 1:**
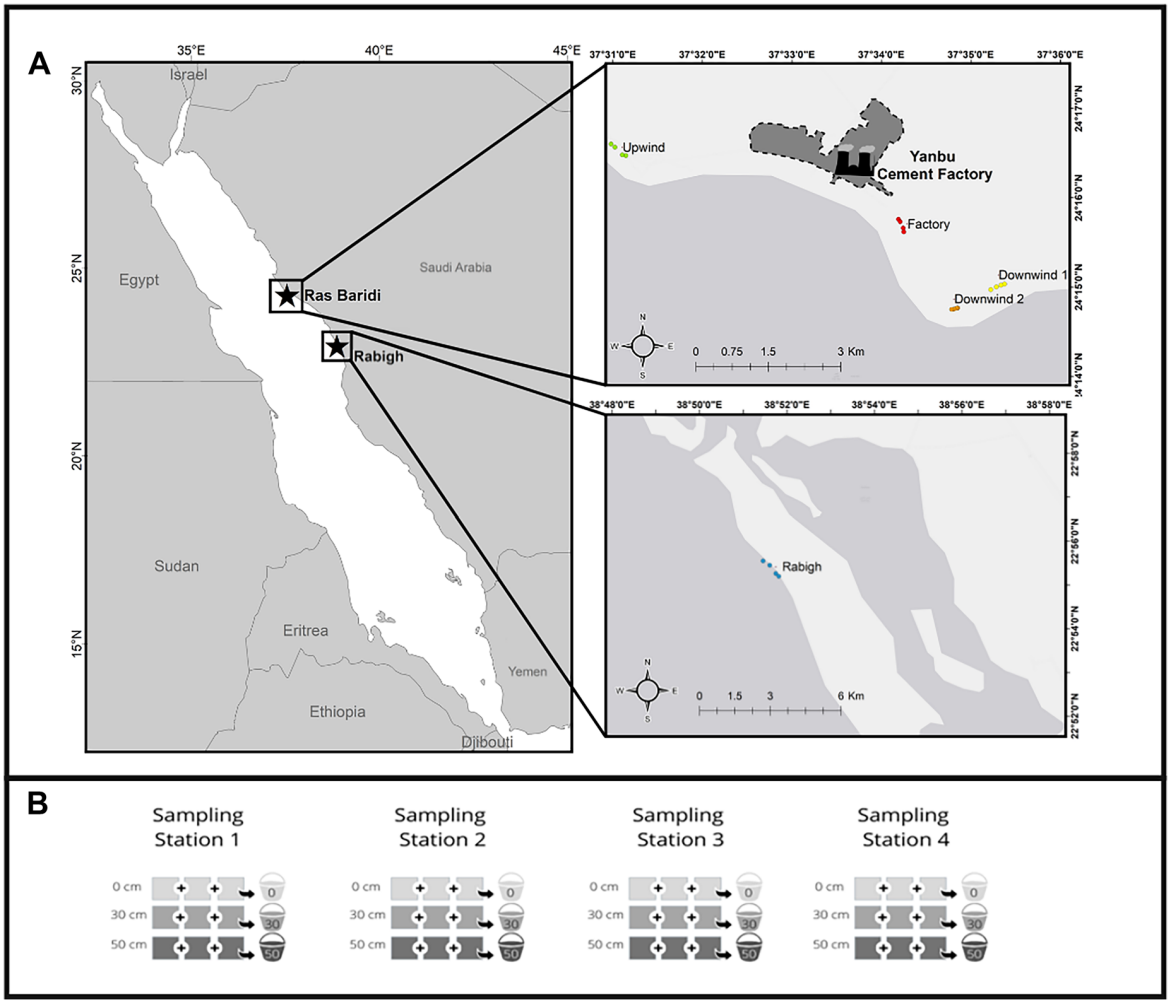
**A** Sand samples for assessments of heavy metal concentrations were collected from two regions along the Saudi Arabian Red Sea, including Ras Baridi, the largest green turtle rookery in Saudi Arabia, and Rabigh, our reference nesting site (blue circles). We sampled four beaches at Ras Baridi, at varying distances from the Yanbu Cement Factory, including Upwind (green), Factory (red), Downwind 1 (yellow), and Downwind 2 (orange). **B** At each of the five beaches studied, four sampling stations were chosen, equally spaced apart. The small colored circles on map **A** represent the different sampling stations at each beach. At each sampling station, three collection points were selected 1 m apart from each other at 0, 30, and 50 cm depth, and homogenized by depth

Sand samples were collected from two regions, Ras Baridi and Rabigh, along the Saudi Arabian coastline of the Red Sea (Fig. 1A). Ras Baridi is Saudi Arabia’s largest green turtle rookery with occasional evidence of hawksbill turtle nesting (Al-Merghani et al., 2000; Shimada et al., 2021). Most nesting occurs on five isolated beaches along a 6-km stretch of coastline at varying distances from the Yanbu Cement Factory, which uses limestone from a nearby fossil reef for its cement production. The coastline is bordered by a fringing coral reef and large seagrass meadows that provide foraging habitats for hawksbill turtles, green turtles, and dugongs (Pilcher & Al-Merghani, 2000; Preen, 1989). In total, we collected sand samples from five turtle nesting beaches, four in Ras Baridi and one in Rabigh, which was used as an uncontaminated reference. In Ras Baridi, four of these beaches were chosen to assess how wind influences the dispersion of heavy metals, as the prevailing wind direction at Ras Baridi was from north to south (Langodan et al., 2017). The beaches at Ras Baridi ranged from 800 m to 5.5 km apart from each other (Fig. 1A). One beach was 5 km upwind from the factory (Fig. 1A, green), one beach was adjacent to the factory (Fig. 1A, red), and two beaches were 3.2 (Fig. 1A, yellow) and 3.5 km (Fig. 1A, orange) from the factory. Henceforth, the sites will be referred to as “Upwind,” “Factory,” “Downwind 1,” and “Downwind 2” (Fig. 1A). The second region sampled, Rabigh Beach, is a coastal beach with evidence of green turtle nesting (Fig. 1A, blue). Although this site is approximately 200 km south of Ras Baridi, it was chosen because there are few known coastal turtle nesting sites in Saudi Arabia for comparison. Rabigh Beach is approximately 11 km long on a sandy peninsula, with most green turtle nesting evidence found at the northern tip of the peninsula. Adjacent to the beach is a fringing coral reef, where hawksbill turtles are often seen foraging and green turtles are occasionally seen resting. The prevalent wind conditions in this region are also from north to south (Langodan et al., 2017). Furthermore, Rabigh has both a cement factory (45 km downwind from the beach) and a petroleum factory (27 km downwind from the beach), but we do not believe that there would be a major influence on nesting sites owing to the distance and wind direction. In addition, Rabigh Beach is less affected by anthropogenic activities as the beach is only accessible by a dirt road, with limited traffic.

At each of the five beaches, we selected four sampling stations spaced equally apart on the nesting beach, maintaining equal distance from the high tide line (Fig. 1B). At each of the four sampling stations, three sand collection points were placed 1 m apart. Using a standardized scoop, 100 ± 10 g of sand was collected from each of the three collection points at the surface (0 cm), average depth of hawksbill turtle nests (30 cm), and average depth of green turtle nests (50 cm). To reduce the impact of natural variability on the composition of the sand in the analyses, three scoops were combined (i.e., approximately 300 g) per sampling depth at each of the four sampling stations per beach (Fig. 1B). The combined 300 g sample (taken from a specific depth at each sampling station) was homogenized and frozen until analysis. Therefore, four samples from each of the three depths were obtained at each beach. Overall, 60 samples (3 depths × 4 sampling stations × 5 beaches) were obtained.

Upon analysis, 100 g of sand was dried at 65 °C in a Binder incubator for 24 h. After drying, each sample was finely ground and homogenized using a porcelain mortar and pestle. For each ground sample, 250 mg was digested with a mixture of 1.5 mL HNO3 and 4.5 mL of 37% HCl using an Ultrawave digestion system. The samples were left to cool before dilution with Milli-Q water. Subsequent analyses were performed using an Agilent 8800 Inductively Coupled Plasma Mass Spectrometer (ICP-MS) to analyze the concentrations of Cd, Pb, Cr, Ni, Se, Sb, As, and Cu. An Agilent 5100/5110 VDV Inductively Coupled Plasma-Optical Emission Spectrometry (ICP-OES) was used to assess the concentrations of Fe. These heavy metals were chosen because they were used in heavy metal monitoring around turtle nesting beaches in other parts of the world (Celik et al., 2006; Kaska et al., 2004; Kaska & Furness, 2001) and are known to cause health problems in turtles and many other organisms (Grillitsch & Schiesari, 2010).

### The influence of wind dispersion on the distribution of heavy metals at turtle nesting beaches near the cement factory

To assess how wind affected the dispersion of heavy metals at Ras Baridi, data were tested for equality of variances using Levene’s test and log transformed, if needed. One-way ANOVAs were then used to assess the concentration of each heavy metal by beach (Upwind, Factory, Downwind 1, Downwind 2). Tukey’s honest significant difference (HSD) test was used to determine the significance of each metal among beaches.

### Distribution of heavy metals along the depth profile of sea turtle nests

All statistical analyses were performed using R version 4.0.3 (R Core Team, 2021), and statistical significance was assumed at *p* < 0.05. First, Levene’s test was used to assess the equality of variances, and the data were log transformed, where necessary. ‍‍‍Factorial analysis of variances (ANOVAs) was performed to evaluate the interactions among the three sand depths (0, 30, and 50 cm) and beaches (Rabigh, Upwind, Factory, Downwind 1, and Downwind 2) for each metal (As, Cd, Cr, Cu, Fe, Ni, Pb, Sb, and Se). After assessing the interaction effects, a one-way ANOVA was conducted to determine the effects of depth on the concentrations of each metal.

### Heavy metal comparison between Ras Baridi and Rabigh Beach

To examine the differences in heavy metal concentrations between the nesting site adjacent to the Yanbu Cement Factory in Ras Baridi and the reference beach in Rabigh, we applied Levene’s test and transformed the necessary data, followed by an independent 2-group *t*-test. We then compared the concentration of each individual heavy metal (As, Cd, Cu, Cr, Ni, Sb, Se, Pb, and Fe).

### Environmental contamination comparison between Ras Baridi and Rabigh Beach

The concentrations of heavy metals that were sampled at the turtle nesting beaches were further analyzed using pollution indices, including the contamination factor (CF) and geo-accumulation index (*I*_*geo*_). Previous studies have used these indices to quantify the risk of heavy metal pollution in the region (e.g., El-Sherbiny et al., 2019; El-Sorogy et al., 2021; Youssef & El-Sorogy, 2016).

The contamination factor (CF) is a single index that is considered an effective tool for monitoring heavy metal contamination (Hakanson, 1980). The CF accounts for contamination by a single element and is defined by the following equation:

$$C{F}_{i}=\frac{{C}_{i}}{{B}_{i}}$$

where *C*_*i*_ is the measured concentration of heavy metal *i*, and *B*_*i*_ is the background value of metal *i* (when there was no anthropogenic input)*.* The contamination factor differentiates four distinct classes (low, moderate, considerable, and very high) based on the CF value (Table S1). When available, we used background heavy metal concentrations from the Red Sea (Ruiz-Compean et al., 2017) and background shale values from Turekian and Wedepohl (1961) when the Red Sea values were unavailable. As there were no significant differences among depths, the average heavy metal concentration for each beach was used for *C*_*i*_.

To quantify the intensity of contamination, Muller (1969) introduced the geo-accumulation index (*I*_*geo*_), which was determined by comparing present-day heavy metal concentrations with the geochemical background (pre-civilized background values). *I*_*geo*_ was calculated using the following equation:

$${I}_{geo}={log}_{2}\ \left[\frac{{C}_{n}}{1.5\times {B}_{n}}\right]$$

where *C*_*n*_ is the concentration of heavy metal *n* measured in the sediment and *B*_*n*_ is the geochemical background value in the upper continental crust of the Earth (Taylor & McLennan, 1995). The constant 1.5 accounts for the variability in the reference value owing to of the effect of lithogenic processes (Muller, 1969). Background values for Cd, Pb, Fe, Cr, Ni, Se, Sb, As, and Cu from the upper continental crust were determined by Taylor and Mclennan (1995). *I*_*geo*_ values are categorized into seven classes, as categorized by Muller (1969), ranging from uncontaminated to extremely contaminated (Table S2). As there were no significant differences between depths, the average heavy metal concentration for each beach was used for *C*_*n*_.

## Results and discussion

### The influence of wind dispersion on the distribution of heavy metals at turtle nesting beaches near the cement factory

**Fig. 2:**
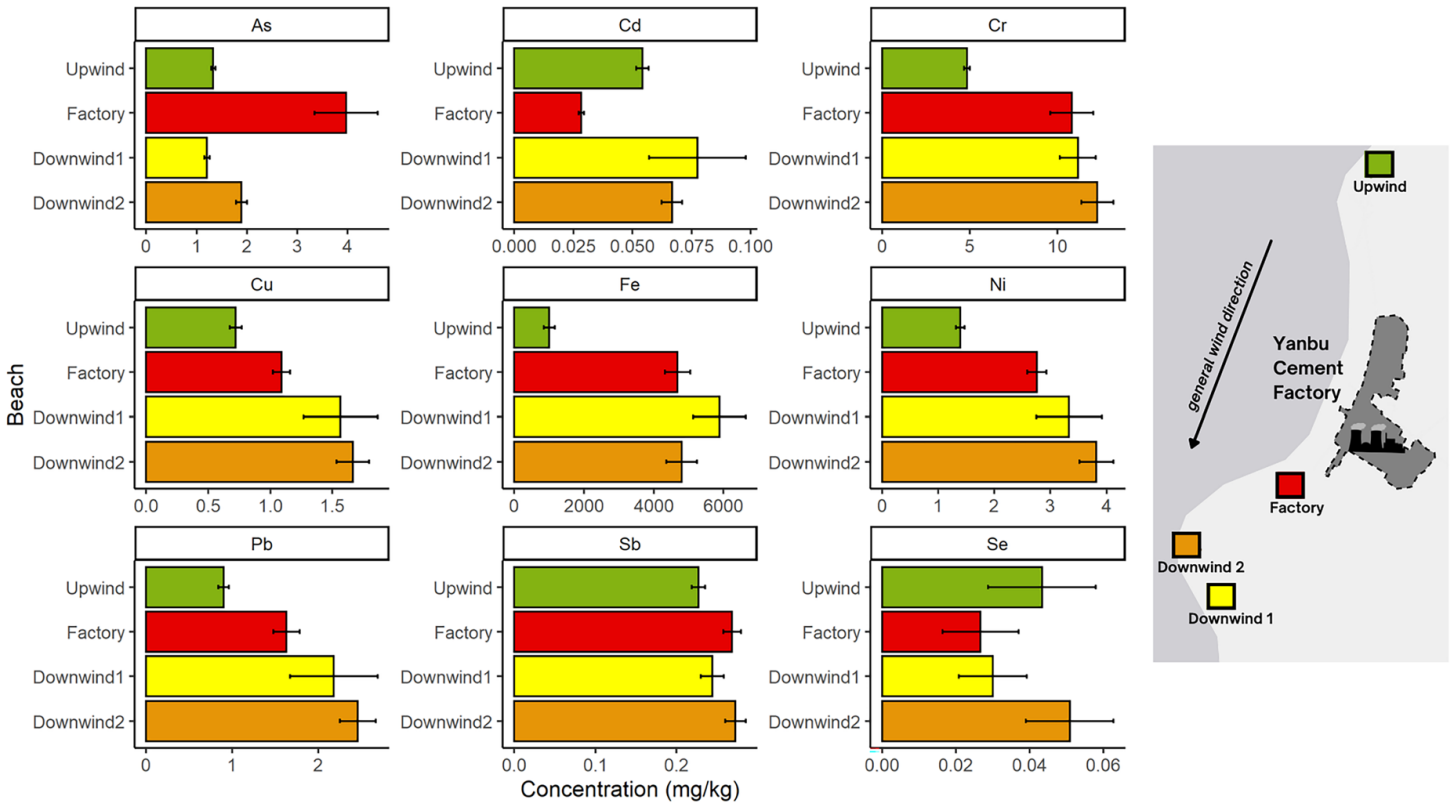
Average heavy metal and metalloid concentrations (mg/kg) ± standard error (se) of arsenic (As), cadmium (Cd), chromium (Cr), copper (Cu), iron (Fe), nickel (Ni), lead (Pb), antimony (Sb), and selenium (Se) from four turtle nesting beaches, varying distances away from a cement factory near Yanbu, Saudi Arabia. All average concentrations are shown on the x-axis (note varying scale for each element). The locations of the four beaches are indicated on the right panel and include Upwind, Factory, Downwind 2, and Downwind 1

The impact of the prevailing wind direction on the distribution of heavy metals at each beach location relative to the Yanbu Cement Factory showed an overall trend of decreasing concentration as follows: Downwind 1, Downwind 2, Factory, and Upwind (Fig. 2). One-way ANOVA tests for each metal (As, Cd, Cr, Cu, Fe, Ni, Pb, Sb, and Se) revealed significant differences in heavy metal concentrations among the beaches, except for Se, which had similar concentrations at each beach (F_4,55_ = 1.664, *p* = 0.172, Table S3). The results supported our hypothesis that heavy metal concentrations would be higher adjacent to and downwind from the cement factory compared to upwind. This finding was similar to that of a study conducted around a cement factory in Rabigh, which found that wind played a significant role in the transfer of pollutants (El-Sherbiny et al., 2019). Future developments should consider the prevailing wind direction and potential sources of pollution relative to nesting areas to maintain important marine habitats (Meylan, 1988; Thayer et al., 2016) and support the conservation of flagship species that are imperative for ecotourism (Tisdell & Wilson, 2002).

Another key conservation implication of our findings was for clutch relocation. There are plans to create a marine protected area at Ras Baridi (Saudi Green Initiative, 2021), including a potential hatchery to incubate relocated clutches from doomed nests, which are nests that would likely face high mortality from natural causes, such as tidal inundation (Martins et al., 2021). Clutch relocation is a common conservation strategy to increase hatching success (García et al., 2003), and the results revealed that clutches should be relocated upwind of the factory to reduce the potential impacts of the cement dust and heavy metal contamination. Additionally, a study found that the emergent success of relocated eggs north of the cement factory (upwind) were significantly higher than that of the beaches downwind (Pilcher, 1999). These findings suggest that the cement dust at Ras Baridi negatively influenced the success of the clutches south of the factory owing to the hardened sediment “shell” formed above the nest (Pilcher, 1999).

### Distribution of heavy metals along the depth profile of sea turtle nests

There was no clear trend between the heavy metal concentration and sampling depth, and there were no significant interactions between the beach and depth for most metals. Factorial ANOVAs for each metal showed no significant interactions between depth and beach for Cd, Cr, Cu, Fe, Ni, or Pb (Table S4). However, we observed significant interactions for As (F_8,45_ = 7.790, *p* < 0.001) and Sb (F_8,45_ = 2.428, *p* = 0.028).

**Fig. 3:**
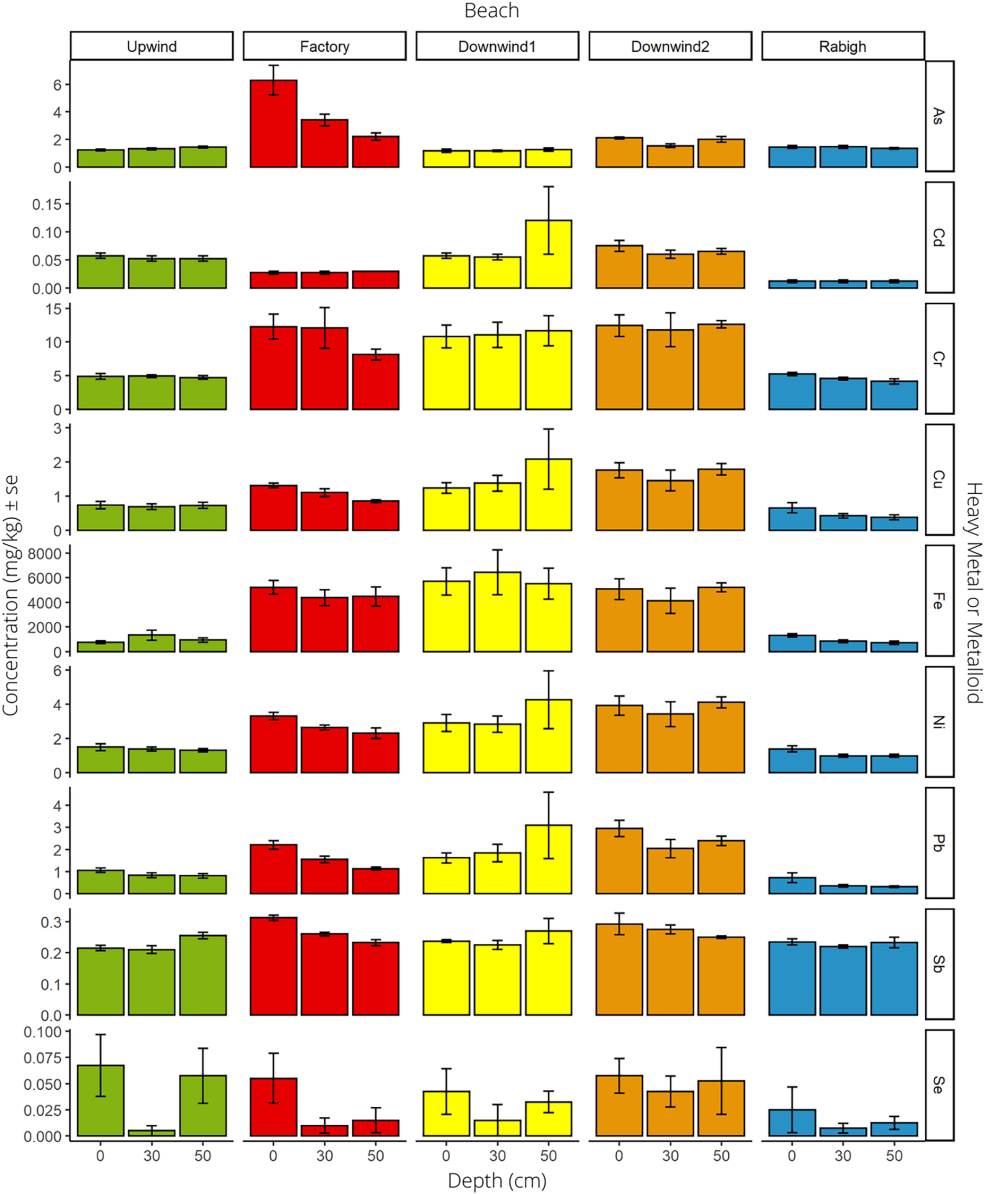
Average heavy metal and metalloid concentrations (mg/kg) ± standard error (se) of arsenic (As), cadmium (Cd), chromium (Cr), copper (Cu), iron (Fe), nickel (Ni), lead (Pb), antimony (Sb), and selenium (Se) from 3 depths, 0, 30, and 50 cm. These depths correspond to sand surface, and the average nest depth of hawksbill and green turtles, respectively. All average concentrations are shown on the left y-axis (note varying scale for each element), and the metals are identified on the right y-axis. Five nesting beaches on the Saudi Arabian Red Sea coast were sampled, including Rabigh Beach (the reference beach) and four beaches (Upwind, Factory, Downwind 1, and Downwind 2) located near the Yanbu Cement Factory in an area known as Ras Baridi

After assessing the interaction effects, we conducted ANOVA tests to compare the differences among depths (0, 30, and 50 cm) for each element. The only metal that showed a significant difference among the three sand depths was Se (F_2,57_ = 4.137, *p* = 0.021; Fig. 3, Table S3). Tukey’s post hoc test showed that Se in surface sand (0 cm) had a significantly higher (*p* = 0.015) concentration (0.050 mg/kg ± 0.010) than that measured at 30 cm (0.016 mg/kg ± 0.005). For all the other metals, there were no significant differences among the three sand depths (Fig. 3).

The surface sand was expected to have higher concentrations of heavy metals because of the cement dust accumulating on the surface of the sand. Furthermore, other studies have found that surface sediments have higher heavy metal concentrations than sub-surfaces (Al-Mur et al., 2017; Bahram et al., 2021). However, our results did not confirm this hypothesis. The sand at Ras Baridi may be more vertically homogenized due to frequent turtle nesting, which mixes sand from different depths. However, Rabigh had a much lower density of nesting turtles, and the heavy metal concentrations were not significantly different by depth. Therefore, a lack of stratification in heavy metal concentrations was found at the two sites; however, further investigation is needed to assess if this is true for all turtle nesting beaches along the Saudi Arabian coast. It is worth noting that this study was conducted in one period, during the green turtle nesting season, which may have promoted the homogenization of heavy metal concentrations in the analyzed nests. Therefore, assessing heavy metal concentrations at various depths during winters, when nesting is less common, may reveal if the homogeneity in metal concentrations is a result of turtle nesting behavior.

### Heavy metal comparison between Ras Baridi and Rabigh Beach

**Fig. 4:**
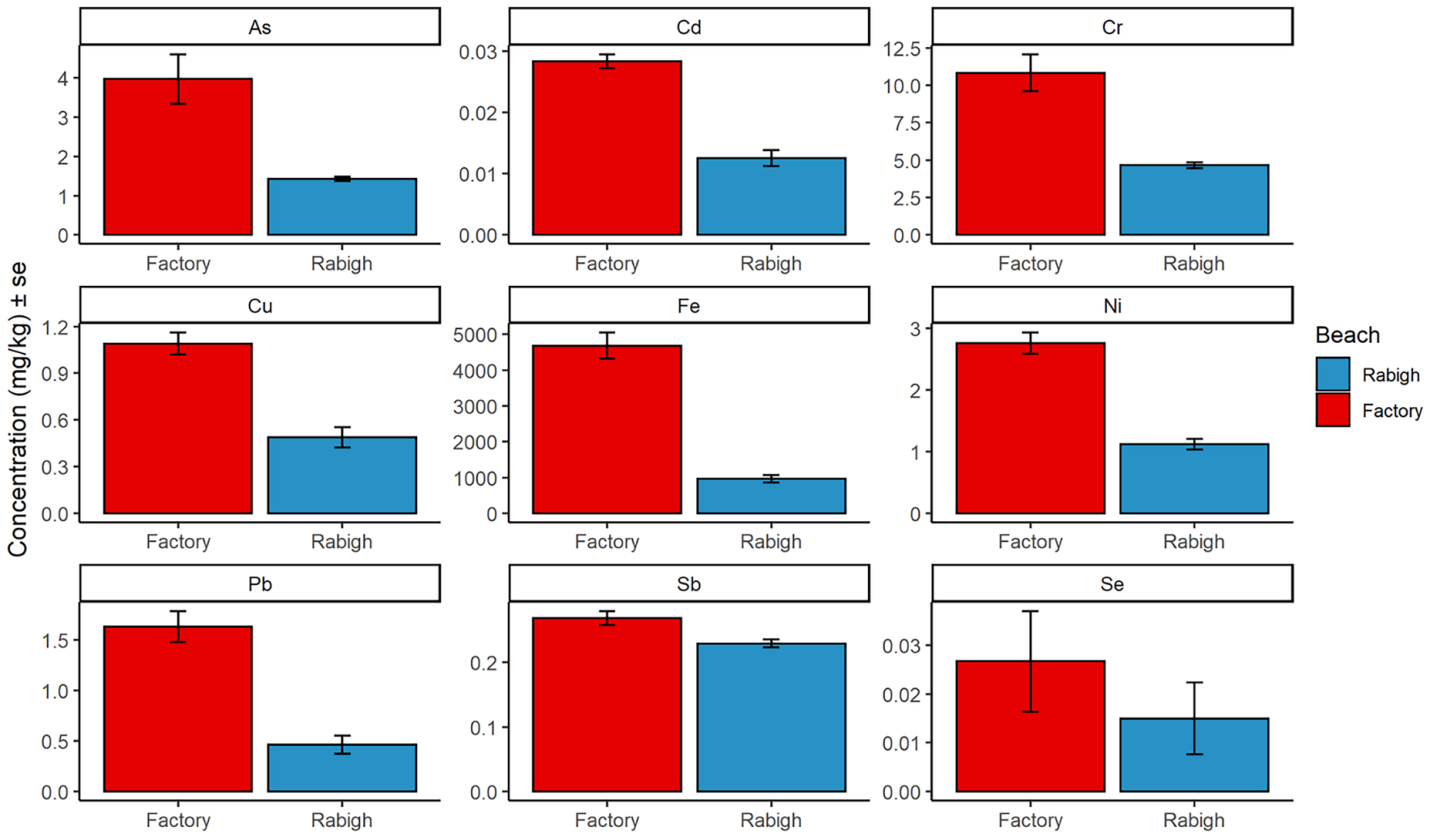
Average heavy metal concentration ± standard error of As, Cd, Cr, Cu, Ni, Pb, Sb, Se, and Fe measured at turtle nesting sites in Saudi Arabia, including a site adjacent to the cement factory in Yanbu (red) and Rabigh Beach, our uncontaminated reference site (blue). An independent 2-group t-test revealed significantly higher concentrations of every element at the factory site compared to the Rabigh Beach site

Heavy metal concentrations were significantly higher at the nesting beach closest to the cement factory at Ras Baridi than at Rabigh Beach, which supported the hypothesis that the cement factory was likely to have an impact on the distribution of heavy metals. The independent 2-group *t*-tests revealed that almost all heavy metals were detected at significantly higher concentrations at the Ras Baridi factory site than at Rabigh Beach, the uncontaminated reference site (Fig. 4, Table S5). The only exception was Se, where no significant difference was found between concentrations measured next to the factory (mean 0.03 ± 0.04 mg/kg) compared to Rabigh Beach (mean 0.02 ± 0.03 mg/kg), t(19.9) = 0.92, *p* = 0.37. Se is found in the environment from both natural and anthropogenic sources, used in glass manufacturing, as an additive to metal alloys, and as a pigment in paints and plastics (Mehdi et al., 2013). Cement production was an industrial source of particulate matter and metals, especially As, Cd, Cu, Ni, and Pb (Chen et al., 2010; Gupta & Sharma, 2013) which could explain why these elements had significantly higher concentrations measured next to the cement factory at Ras Baridi compared to Rabigh Beach (As, t(15.53) = 7.70, *p* < 0.001; Cu, t(21.87) = 6.37, *p* < 0.001; Cd, t(21.52) = 9.12, *p* < 0.001; Ni, t(16.34) = 8.46, *p* < 0.001; and Pb, t(17.75), *p* < 0.001).

Cr was another heavy metal detected at significantly higher concentrations at the factory site than at Rabigh Beach (t(15.23) = 7.31, *p* < 0.001; Fig. 4). Sources of Cr in the environment include coal-fired power production, mineral ore and petroleum refining, fuel combustion, and cement production (Choppala et al., 2013). Chromium was measured higher downwind and adjacent to the factory compared to upwind (Fig. 3), suggesting that the cement dust could be a source, as Cr can be present in the raw materials used in cement production (Eštoková et al., 2012). Iron concentrations were also significantly higher at the factory site than at Rabigh Beach (t(20.29) = 11.99, *p* < 0.001; Fig. 4). Iron is found in high concentrations naturally in the Earth’s crust (EPA, 1993). The concentration measured upwind from the factory was found to be much lower than all the other sites at Ras Baridi (Fig. 3); therefore, it was possible that Fe was also transported in the wind from the cement factory. Lastly, higher concentrations of Sb were found at the factory site than at Rabigh Beach (t(17.90) = 3.10, *p* = 0.006; Fig. 4). It enters the environment from burning coal, mines, and industrial facilities and is used frequently as a flame retardant (Filella et al., 2002). Because the concentrations were similar at all the sites at Ras Baridi (Fig. 3), it was possible that this element was not transferred in the wind, but occurred naturally in the sediment (Filella et al., 2002), or was transferred from the nearby road, as Sb is used in brake linings (Földi et al., 2018).

Future studies should be conducted to investigate how heavy metal concentrations compare at Ras Baridi between freshly laid eggs and hatchlings to assess whether heavy metals accumulate over time through direct contact with contaminated sand (Al-Rawahy et al., 2007). This study emphasized the potential for pollution from cement factories to affect other trophic levels. Many animals consume turtle hatchlings as prey and metals like Pb and Cd could potentially be transferred through the food web (Kaska & Furness, 2001). Many animals consume turtle hatchlings as prey and these metals could potentially transfer through the food web. For example, Arabian foxes (*Vulpes vulpes arabica*) in the Ras Baridi region consume turtle eggs and hatchlings (Pilcher, 1999). Furthermore, future studies should focus on heavy metal concentrations inside the seagrass meadows found in the shallow waters around the Yanbu Cement Factory, as dugongs and turtles were recorded grazing there (Preen, 1989). A 1995 study found that essential elements, including Fe, Mn, Zn, and Cu, are easily transferred from mother to eggs, with a limited transfer of toxic metals, including Cd and Hg (Sakai et al., 1995; 2000). Thus, any female turtles foraging in this area could accumulate heavy metal contaminants, potentially passing them off to their offspring.

### Environmental contamination comparison between Ras Baridi and Rabigh Beach

An assessment of the environmental contamination of the five turtle nesting beach samples revealed that 68% of the estimated CFs were less than one, indicating that the average concentrations recorded from each beach were not higher than the background levels (Table 1). The CF values calculated for Rabigh Beach were relatively low (Hakanson, 1980), which was expected owing to its distance from cities and human impact (Velea et al., 2009).

**Table 1:**
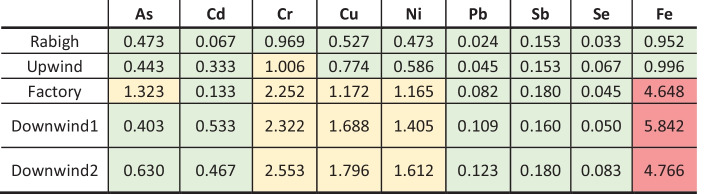
Contamination factor (CF) based on average heavy metal concentrations measured at five nesting beaches on the Saudi Arabian Red Sea coast. Upwind, Factory, Downwind 1, and Downwind 2 located in close proximity to a cement factory near Yanbu, and Rabigh Beach is a reference site 200 km south of Yanbu. Values were categorized in different levels of contamination from Hakanson (1980). Values in green have low contamination, values in yellow have moderate contamination, and values in red have considerable contamination

Fe had the highest CF values, in the “considerable contamination” category (Hakanson, 1980). While important for the growth and function of many living organisms (Valko et al., 2005), the elevated Fe levels were not found at the site upwind from the cement factory or Rabigh Beach; suggesting that the contamination could have originated from the cement dust. Although Fe is an essential element, it can be toxic at high concentrations (Madiwale & Liebelt, 2006). Some beaches showed moderate contamination with Cr, Cu, and Ni, with an increasing trend in the following order: CF_Upwind_ < CF_Factory_ < CF_Downwind 1_ < CF_Downwind 2_. Furthermore, the beach adjacent to the cement factory was the only site with elevated CF values of As, suggesting moderate contamination (Hakanson, 1980). A study found that elevated levels of As, Cr, Cu, and Ni could lead to many health effects in turtles, including cancer, reproductive and developmental disorders, endocrine disruption, immune function disorders, renal and hepatic dysfunction, and neurotoxic disorders (Grillitsch & Schiesari, 2010). Additionally, the background shale values for Se, Sb, and Pb were unavailable for this region (Ruiz-Compean et al., 2017); therefore, global values were used (Turekian & Wedepohl, 1961); thus, the CF values for these elements might not be as accurate as those of the other elements.

According to the geo-accumulation index, most of the sites included in this study were uncontaminated (Table 2). Additionally, the beach closest to the factory showed slightly elevated *I*_*geo*_ As levels, classified as “uncontaminated to moderately contaminated” (Muller, 1969). Marco et al. (2004) tested whether arsenic could permeate through the flexible eggshells of reptiles during incubation. They found that in As-contaminated substrates, eggs absorbed As, resulting in embryos that accumulated considerable amounts of As during incubation. In addition, hatchlings incubated in As-contaminated substrate had reduced running speeds, indicative of hindered escape and foraging efficiency, which ultimately influenced the survival and success of individuals (Marco et al., 2004). Although none of the elements in this study had CF or *I*_*geo*_ values classified as high risk (Hakanson, 1980), this does not distract from the impact of heavy metals on the ecology around the cement factory, and future studies should further assess this issue.

**Table 2:**
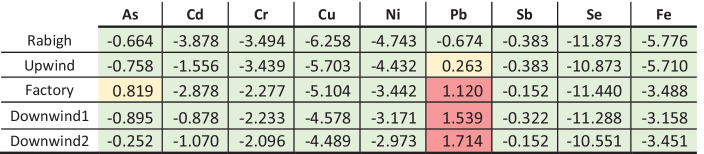
Geo-accumulation index (I_geo_) based on average heavy metal concentrations measured at five nesting beaches on the Saudi Arabian Red Sea coast. Upwind, Factory, Downwind 1, and Downwind 2 located in close proximity to a cement factory near Yanbu, and Rabigh Beach is a reference site 200 km south of Yanbu. Values were categorized in different levels of contamination from Muller (1969). Values in green are classified as uncontaminated, values in yellow are uncontaminated to moderately contaminated, and values in red are moderately contaminated

Lead also showed elevated geo-accumulation levels, which followed an increasing trend of *I*_*geo*_(Pb) values: *I*_*geo*_(Pb)_Upwind_ < *I*_*geo*_(Pb)_Factory_ < *I*_*geo*_(Pb)_Downwind1_ < *I*_*geo*_(Pb)_Downwind2_. The *I*_*geo*_(Pb) value was considered “uncontaminated to moderately contaminated” at the Upwind location, whereas Factory, Downwind 1, and Downwind 2 sites had levels considered “moderately contaminated” (Muller, 1969). Lead, a naturally occurring metal in the Earth’s crust, is now a globally distributed pollutant as it was commonly added to gasoline (Mao et al., 2009). Burger et al. (1998) examined the effects of Pb on the behavioral development of slider turtle hatchlings (*Trachemys scripta*) and found that survival declined as a function of Pb dosage. The self-righting response time and hatchling morphology were also influenced by Pb (Burger et al., 1998). Because the heavy metal concentrations observed in our study have the potential to affect the local population of sea turtles (Grillitsch & Schiesari, 2010), additional studies focusing on the impact of these heavy metals on sea turtles should be conducted.

## Conclusion

Overall, higher heavy metal concentrations were found at the Ras Baridi beaches than at Rabigh Beach, which was expected due to its proximity to the cement factory. Furthermore, we found that the four nesting beaches in Ras Baridi had significantly different heavy metal concentrations. The nesting beach upwind from the factory had lower levels than downwind. The sampling depth was not found to have a significant effect on the concentrations of most of the heavy metals. In addition, the contamination factor and geo-accumulation index were not at high risk for any element at the study sites; however, we want to highlight that this does not imply that the heavy metals near the cement factory do not influence the nearby ecology. Under “Vision 2030,” the Kingdom of Saudi Arabia plans to diversify its economy through tourism. This includes several giga-projects planned for the nation, many of which are located along the Red Sea coast (PIF, 2017). Therefore, there are risks of rapid coastal development, increased urbanization, and the expansion of industrial activities, which could increase heavy metal pollution. Due to the upcoming large-scale tourism development projects planned on the Saudi Arabian coast, heavy metal monitoring should be used at turtle nesting sites to assess human-caused pollution on turtle nesting beaches.

## Supplementary Information

Below is the link to the electronic supplementary material.

Supplementary file1 (DOCX 19 KB)

## Data Availability

The datasets generated and/or analyzed during the current study are available from the corresponding author upon request.
