# Supplementary material for: Potential effects of heavy metal pollution from a cement factory near Saudi Arabia’s largest green turtle rookery
Source: Environ Monit Assess. 2022 May 24;194(6):450. doi: 10.1007/s10661-022-10063-2 (PMC9130198; doi:10.1007/s10661-022-10063-2)
Supplement: Supplementary file 1 — Supplementary file1 (DOCX 19 KB) [file 10661_2022_10063_MOESM1_ESM.docx]

**Potential effects of heavy metal pollution from a cement factory near Saudi Arabia’s largest green turtle rookery**

Lyndsey K. Tanabe^1^, Susana Carvalho^1^, Vijayalaxmi Dasari^1^, Areen Nasif^1^, Kaitlyn A. O’Toole^1^, & Michael L. Berumen^1^

*^1^Red Sea Research Center, Division of Biological and Environmental Science and Engineering, King Abdullah University of Science and Technology, Thuwal, 23955, Saudi Arabia*

**Correspondence:** [Lyndsey.Tanabe@kaust.edu.sa](mailto:Lyndsey.Tanabe@kaust.edu.sa)

**Table S1**. Classes based off Contamination Factor (CF) index (Hakanson, 1980).

| **Contamination Factor (CF)** | **Level of Contamination** |
| --- | --- |
| CF < 1 | Low contamination |
| 1 < CF < 3 | Moderate contamination |
| 3 < CF < 6 | Considerable contamination |
| CF > 6 | Very high contamination |

**Table S2**. Sediment quality classifications based off Muller (1969) Geoaccumulation Index (Igeo).

| **Igeo** | **Sediment class** | **Sediment quality classification** |
| --- | --- | --- |
| 0 | 0 | Uncontaminated |
| 0-1 | 1 | Uncontaminated to moderately contaminated |
| 1-2 | 2 | Moderately contaminated |
| 2-3 | 3 | Moderately to strongly contaminated |
| 3-4 | 4 | Strongly contaminated |
| 4-5 | 5 | Strongly to extremely contaminated |
| >5 | 6 | Extremely contaminated |

**Table S3**. Significance table for ANOVA comparing differences between depths (0, 30, and 50 cm) and beaches (Rabigh, Upwind, Factory, Downwind1, and Downwind2) for each metal (arsenic (As), cadmium (Cd), chromium (Cr), copper (Cu), iron (Fe), nickel (Ni), lead (Pb), antimony (Sb), and selenium (Se))*.* Significance was determined if *p* < 0.05.

| **Metal** | **Depth** | | **Beach** | |
| --- | --- | --- | --- | --- |
|  | *F* | *p* | *F* | *p* |
| As | 1.895 | 1.895 | 16.04 | < 0.001 |
| Cd | 0.688 | 0.688 | 8.179 | < 0.001 |
| Cr | 0.210 | 0.811 | 35.84 | < 0.001 |
| Cu | 0.287 | 0.287 | 11.12 | < 0.001 |
| Fe | 0.048 | 0.048 | 63.18 | < 0.001 |
| Ni | 0.349 | 0.349 | 14.72 | < 0.001 |
| Pb | 0.557 | 0.557 | 10.38 | < 0.001 |
| Sb | 1.252 | 1.252 | 3.896 | 0.007 |
| Se | 4.137 | 0.021 | 1.664 | 0.172 |

**Table S4**. Significance tables for Factorial ANOVAs assessing interaction effects between depth (0, 30, and 50 cm) and beach (Rabigh, Upwind, Factory, Downwind1, and Downwind2) for each metal (arsenic (As), cadmium (Cd), chromium (Cr), copper (Cu), iron (Fe), nickel (Ni), lead (Pb), antimony (Sb), and selenium (Se)). Significance was determined if *p* < 0.05.

| **Metal** | **Depth** | | **Beach** | | **Interaction** | |
| --- | --- | --- | --- | --- | --- | --- |
|  | *F* | *p* | *F* | *p* | *F* | *p* |
| As | 6.427 | 0.004 | 69.651 | < 0.001 | 7.790 | < 0.001 |
| Cd | 1.051 | 0.358 | 8.309 | < 0.001 | 1.097 | 0.383 |
| Cr | 0.447 | 0.642 | 17.919 | < 0.001 | 0.559 | 0.805 |
| Cu | 0.484 | 0.619 | 10.844 | < 0.001 | 0.961 | 0.478 |
| Fe | 0.471 | 0.627 | 63.952 | < 0.001 | 1.216 | 0.312 |
| Ni | 0.654 | 0.525 | 13.988 | < 0.001 | 0.746 | 0.651 |
| Pb | 0.961 | 0.39 | 10.794 | < 0.001 | 1.282 | 0.277 |
| Sb | 1.798 | 0.177 | 5.335 | 0.00133 | 2.428 | 0.028 |
| Se | 4.043 | 0.0243 | 1.721 | 0.162 | 0.477 | 0.866 |

**Table S5.** Results from independent 2-group t-test, which was used to compare each heavy metal concentration (arsenic (As), cadmium (Cd), chromium (Cr), copper (Cu), iron (Fe), nickel (Ni), lead (Pb), antimony (Sb), and selenium (Se)), between the beach adjacent to the Yanbu Cement Factory and Rabigh Beach, which was used as a reference.

| **Metal** | **Mean concentration (mg/kg) from Factory** | **Mean concentration (mg/kg) from Rabigh** | **t** | **df** | ***p*** |
| --- | --- | --- | --- | --- | --- |
| As | 3.97 | 1.424 | 7.698 | 17.528 | < 0.001 |
| Cd | 0.028 | 0.013 | 9.192 | 21.523 | < 0.001 |
| Cr | 10.826 | 4.656 | 7.305 | 15.232 | < 0.001 |
| Cu | 1.091 | 0.488 | 6.365 | 21.868 | < 0.001 |
| Fe | 4680.517 | 958.323 | 11.994 | 20.286 | < 0.001 |
| Ni | 2.756 | 1.119 | 8.456 | 16.335 | < 0.001 |
| Pb | 1.631 | 0.466 | 6.567 | 17.747 | < 0.001 |
| Sb | 0.268 | 0.229 | 3.101 | 17.902 | 0.006 |
| Se | 0.027 | 0.015 | 0.921 | 19.850 | 0.368 |
